# *NTRK* fusions and concomitant immune and genomic landscape detected by DNA and RNA comprehensive genomic profiling in a large healthcare system

**DOI:** 10.3389/fmed.2026.1764392

**Published:** 2026-04-02

**Authors:** Alexa K. Dowdell, Thomas R. Ward, Lauren T. Hamilton, Roshanthi K. Weerasinghe, Erica Schnettler, Pratheesh Sathyan, Alison Roos, Josiah T. Wagner, John Welle, Ryan C. Meng, Alexandra Q. Bartlett, Carlo B. Bifulco, Brian D. Piening

**Affiliations:** 1 Earle A. Chiles Research Institute, Portland, OR, United States; 2 Providence St. Joseph Health, Portland, OR, United States; 3 Illumina, Inc, San Diego, CA, United States

**Keywords:** actionability, fusion, NTRK, pan-cancer, real world evidence (RWE)

## Abstract

**Introduction:**

The use of next-generation sequencing (NGS) in clinical investigations has enabled the identification of actionable biomarkers across tumor histologies, paving the way for the development of pan-tumor therapies. Gene fusions involving *NTRK1, NTRK2*, and *NTRK3 (NTRK1/2/3)* have emerged as rare yet clinically significant oncogenic drivers in a wide range of both pediatric and adult tumors due to high response rates to FDA-approved targeted therapies. Consequently, widespread testing for *NTRK* fusions is recommended across tumor types. However, data on *NTRK* fusions in cancer have predominantly been sourced from academic institutions and reference laboratories.

**Methods:**

In this study, we investigated the frequency of *NTRK* fusions and co-occurring genomic alterations across solid tumor types in a large, real-world patient cohort that received DNA and RNA hybrid capture-based comprehensive genomic profiling (CGP) in the Providence community health system.

**Results:**

Among 15,128 adult patients, CGP identified 30 pathogenic *NTRK1/2/3* fusions, corresponding to a clinically actionable prevalence of 0.2% across 12 solid tumor types. An additional 11 *NTRK* fusions were classified as variants of unknown significance, and 8 of the identified *NTRK* fusions in the cohort were novel. The number of distinct and novel fusion partners identified demonstrates the genomic diversity of *NTRK* fusions observed in routine clinical practice.

**Discussion:**

These findings highlight the value of RNA-based NGS, particularly when used alongside DNA NGS, to provide a comprehensive assessment of *NTRK* fusions and co-occurring gene alterations. Implementation of combined DNA and RNA CGP in a community health system setting enables detection of both known and novel *NTRK* fusions and can inform clinical care of cancer patients.

## Introduction

The incorporation of next-generation sequencing (NGS) into translational research and clinical care in oncology has improved the understanding of the genomic drivers of cancer and has enabled more patient-centric treatment approaches (1, 2). Through large-scale NGS studies, actionable biomarkers have been identified across multiple tumor types, leading to the landmark approvals of tumor-agnostic therapies (2–5). To date, there are six pan-tumor genomic biomarkers with FDA approved therapies, and the number is anticipated to grow as additional studies are ongoing (6).

*NTRK1, NTRK2* and *NTRK3* encode the family of TRK receptors that regulate neuronal tissue development and differentiation (7, 8). Gene fusions involving *NTRK1/2*/*3* can act as oncogenes via constitutive activation of downstream signaling pathways that promote cell growth, including the MAPK, PI3K and PKC pathways (9, 10). *NTRK* fusions were first identified in colorectal (CRC) and papillary thyroid cancer (PTC) and have since been detected across multiple adult and pediatric tumors (8). *NTRK* fusions can occur at rates as high as 90% in rare cancers, including infantile fibrosarcoma and mammary analog secretory carcinoma (MASC) (7). *NTRK* fusions are less prevalent in common tumors, normally occurring at frequencies of < 1% in breast, lung, colorectal, melanoma, pancreatic, and brain tumors. *NTRK* fusions are typically mutually exclusive of other genomic driver mutations (11, 12).

*NTRK* fusions were the first gene-specific alteration to have a pan-tumor therapy approval (6). Larotrectinib and entrectinib are first-generation TRK tyrosine kinase inhibitors that are FDA-approved for pediatric and adult patients with *NTRK* fusion-positive metastatic/unresectable solid tumors that have progressed on prior therapies or when no other suitable treatment is available (13). Larotrectinib was approved based on the high overall response rate (75%) in a total of 55 patients with 17 different cancer types harboring *NTRK* fusions (14). Expanded analyses of a broader cohort of patients (*n* = 153) revealed 79% (95% CI, 72–85) of patients with *NTRK*-positive tumors had a response to larotrectinib, with 16% having complete responses (15). Entrectinib was approved based on a pooled analysis of three trials involving 54 adults with advanced or metastatic *NTRK* fusion-positive solid tumors across 10 different tumor types, showing an objective response of 57% (95% CI, 43.2–70.8) (16). Updated integrated analysis of 121 adults with 14 tumor types revealed 61.2% of patients had a complete or partial response, and the median duration of response was 20.0 months (95% CI, 13.0–38.2) (17).

Various clinical laboratory techniques are available to identify gene fusions; however, these vary in sensitivity for *NTRK* fusion detection (18–20). Methods that can be used to detect chimeric *NTRK* fusion proteins include immunohistochemistry (IHC), fluorescence *in situ* hybridization (FISH), reverse transcription polymerase chain reaction (RT-PCR), and next-generation sequencing (NGS) using DNA or RNA (21). FISH, RT-PCR, and IHC have been successfully utilized to detect *NTRK* fusions and are especially useful for initial screening due to their low cost and fast turn-around times. While FISH can detect novel fusions with break apart probes and IHC can detect abnormal expression regardless of partner, these tests are commonly limited to the detection of single biomarkers and are less likely or unable to identify novel *NTRK* fusion partners (20, 21). In addition, sensitivity of FISH may be affected by the presence of non-canonical fusion breakpoints and IHC sensitivity and specificity may be affected by tissue type or which *NTRK* gene is involved (22). Hybrid capture-based RNA sequencing is specifically useful for the detection of novel and known fusion partners and renders a more comprehensive evaluation of clinically actionable fusion genes like *NTRK* compared to targeted and DNA-only NGS techniques (19). Comprehensive genomic profiling assays (CGP) using hybrid-capture based DNA and RNA NGS enable the analysis of all types of genomic alterations found in cancer, including single nucleotide variants (SNVs), indels, splice variants, copy number variants (CNVs), and known and novel gene fusions in hundreds of cancer-related genes. CGP assays also test for immunotherapy biomarkers that rely on the analysis of multiple loci, such as tumor mutational burden (TMB) and microsatellite instability (MSI).

The spectrum of *NTRK* gene fusions observed in clinical practice is not yet completely defined as the number of characterized 5′ fusion partners will likely increase with the adoption of genomic profiling in routine oncology care. Studies of *NTRK* fusions in cancer have mainly come from academic medical centers and reference labs (11, 12, 23). Here, we employed RNA and DNA hybrid capture based CGP to investigate *NTRK* fusions and co-occurring genomic alterations in a large, real-world patient cohort from a community health system.

## Methods and materials

### Cohort selection and assays

Patients in the Providence community health system diagnosed with solid tumors that received CGP testing (*N* = 15,128) as part of their clinical care from August 2018 through September 2023 were included in the analysis. *NTRK1/2/3* fusions had to be reported on the final CGP report as pathogenic or variant of unknown significance (VUS) to be included in *NTRK* cohort. CGP testing was carried out using the ProvSeq 523 or TST170 laboratory-developed procedures (LDPs) that were validated based on College of American Pathologists (CAP) standards. ProvSeq 523 detects DNA variants in 523 genes and RNA fusions in 55 genes from formalin-fixed, paraffin-embedded (FFPE) samples. *NTRK1/2/3* fusions were detected using the RNA portion of the assay. The ProvSeq 523 assay was developed using TruSight™ Oncology 500 High Throughput research reagents (Illumina, San Diego, CA, United States) and sequenced on a NovaSeq 6000 sequencer (Illumina, San Diego, CA, United States). The TST170 LDP utilizes the same ProvSeq 523 assay yet was clinically subset for genes in the TruSight™ Tumor 170 assay (Illumina, San Diego, CA, United States), which covers DNA variants in 148 genes and RNA fusions in 55 genes. This was due to prior limitations on billing and ordering that occurred during the transition from the TruSight™ Tumor 170 assay to the ProvSeq 523 assay in clinical offerings. For research purposes all 523 DNA/RNA genes in the assay were included for patients reported to have a TST170 test (*n* = 2).

### ProvSeq 523/TST170 informatics

Post-sequencing data from both ProvSeq 523 and TST170 assays were processed using the TruSight™ Oncology 500 Analysis Pipeline on Illumina's DRAGEN Bio-IT platform, which converts raw BCL files to FASTQ format, performs hardware-accelerated alignment to the human reference genome, and executes TSO500-specific variant calling for SNVs, indels, CNVs, gene fusions, and splice variants. Key immuno-oncology biomarkers are also computed in the process like tumor mutational burden (TMB) and microsatellite instability (MSI). Resulting VCFs, fusion calls, CNV data, and biomarker outputs are then uploaded to our cloud platform. The annotated variant files are filtered using canonical transcripts and OncoKB data and undergo a secondary round of filtering to restrict to only known/likely pathogenic variants by our team of lab technicians using an in-house variant annotation platform NGSReviewer. Within NGSReviewer, geneticists filter variants based on quality control metrics, literature review and genetic data. Once all variants for a given case have been classified, the pathogenic and VUS variants are passed into a final review platform where molecular pathologists make final determinations on variant pathogenicity and match clinically significant variants with approved medications and potential trials before returning a complete report to the requesting physician.

### ProvSeq 523 validation data and PD-L1 assessment

ProvSeq 523 has been validated and approved by MolDX^®^. The MolDX^®^ program establishes reimbursement rates for molecular diagnostic tests to ensure they are reasonable and necessary for Medicare coverage. In addition, submitted assays are required to pass technical assessments and demonstrate clinical utility. The validation included an analysis of 80 FFPE solid tumor samples for RNA variants including fusions, which had previously been analyzed by the Foundation Medicine CDX assay, Illumina TruSight™ Tumor 170 gene assay, Oncomine Focus RNA assay, ALK gene FISH assay or ROS1 gene FISH assay. The validation data resulted in 100% clinical specificity, 98.75% clinical sensitivity, 99.94% clinical accuracy, a 99.94% negative predictive value (NPV) and 100% positive predictive value. Four known *NTRK* fusions were included in the test samples (*ETV6-NTRK3, LMNA-NTRK1, TGF-NTRK1, TPM3-NTRK1*) and the RNA fusion limit of detection was determined to be 7 fusion copies per ng of RNA. Furthermore, the TruSight™ Oncology 500 assay underlying ProvSeq 523 has recently received FDA approval specifically for the detection of *NTRK* fusions.

PD-L1 was assessed via immunohistochemistry using the Ventana PD-L1 (SP263) assay. PD-L1 positivity was defined as the proportion of tumor cells (TC) and tumor-infiltrating immune cells (IC) expressing PD-L1 ≥ 1%.

### Known vs. novel fusion partner identification

Using data from deidentified electronic medical records and CGP results, *NTRK1/2/3* fusions were assessed in patients with cancer. The Catalog of Somatic Mutations in Cancer (COSMIC) database and a systematic literature review were used to verify novel *NTRK* gene fusion partners (April 2024) with manual literature review to follow (July 2025).

### Assessment for therapeutic actionability

*NTRK* fusions were evaluated for therapeutic actionability and eligibility for TRK inhibitor treatment using public databases (OncoKB and previously IBM Watson and N-of-One) and by expert review. Actionability was determined by the following criteria: the fusion preserved the reading frame of *NTRK*, included the tyrosine kinase domain, and was included on the Providence CGP testing report as a pathogenic *NTRK* gene fusion. Fusions that did not meet these criteria (e.g., fusions resulting in a frameshift of the reading frame, incomplete or complete lack of the tyrosine kinase domain, or additional testing could not confirm the fusion) were marked as a variant of unknown significance (VUS) and reported on the final CGP report as VUS (*n* = 11). *NTRK* fusions reported as VUS were deemed not therapeutically actionable but still included in the cohort as our team was confident in the existence of the *NTRK* fusion. Any *NTRK* fusions that were excluded from the report were not included in the *NTRK* cohort but are still represented in the larger population for a given tumor type. Discretionary supplemental testing using pan-TRK IHC was used to verify actionability when deemed appropriate by a molecular pathologist. As this decision is made on a case-by-case basis, secondary testing was not performed uniformly across all cases. Instances typically include when a novel fusion partner was identified, the fusion had borderline or suboptimal fusion supporting read evidence, and/or the fusion was unexpected for the particular tumor type.

Therapeutic actionability was evaluated using OncoKB Therapeutic Levels of Evidence (Levels 1–4), which are routinely employed in-house as OncoKB serves as our clinical annotation provider. OncoKB Levels 1 and 2 correspond to FDA-recognized and standard-of-care biomarkers predictive of response to FDA-approved therapies, and these categories map directly to AMP/ASCO/CAP Tier I variants of strong clinical significance with Level A evidence. OncoKB Levels 3 and 4 represent biomarkers associated with investigational or context-dependent therapies, including Level 3A biomarkers with compelling clinical evidence for investigational agents, Level 3B biomarkers predictive of response to FDA-approved therapies used off-indication, and Level 4 biomarkers with strong biological but limited clinical evidence; these categories correspond to AMP/ASCO/CAP Level B, C, and D evidence, respectively.

All pathogenic *NTRK* fusions are currently considered actionable with OncoKB Level 1 evidence, reflecting the availability of FDA-approved tumor-agnostic TRK inhibitors, including entrectinib, larotrectinib, and repotrectinib, for solid tumors harboring *NTRK* fusions. Therapeutic actionability of co-occurring mutations was assessed specifically for OncoKB Levels 1 and 2 due to their designation as high-confidence, clinically actionable alterations and their established scope of FDA-approved therapies and standard-of-care treatment options. One patient (#17) whose DNA portion of ProvSeq 523 failed due to input quantity not sufficient (QNS) was removed from co-occurring alteration analyses and therefore is not included in the subsequent heatmap of co-occurring alterations across actionable patients. Statistical analysis was performed in R using the fisher.test() function from package *stats*. A two-sided Fisher's Exact Test was used to test for differences in TMB and MSI between patients with *NTRK* fusions and patients who did not.

### Figure generation

**Figure 1:**
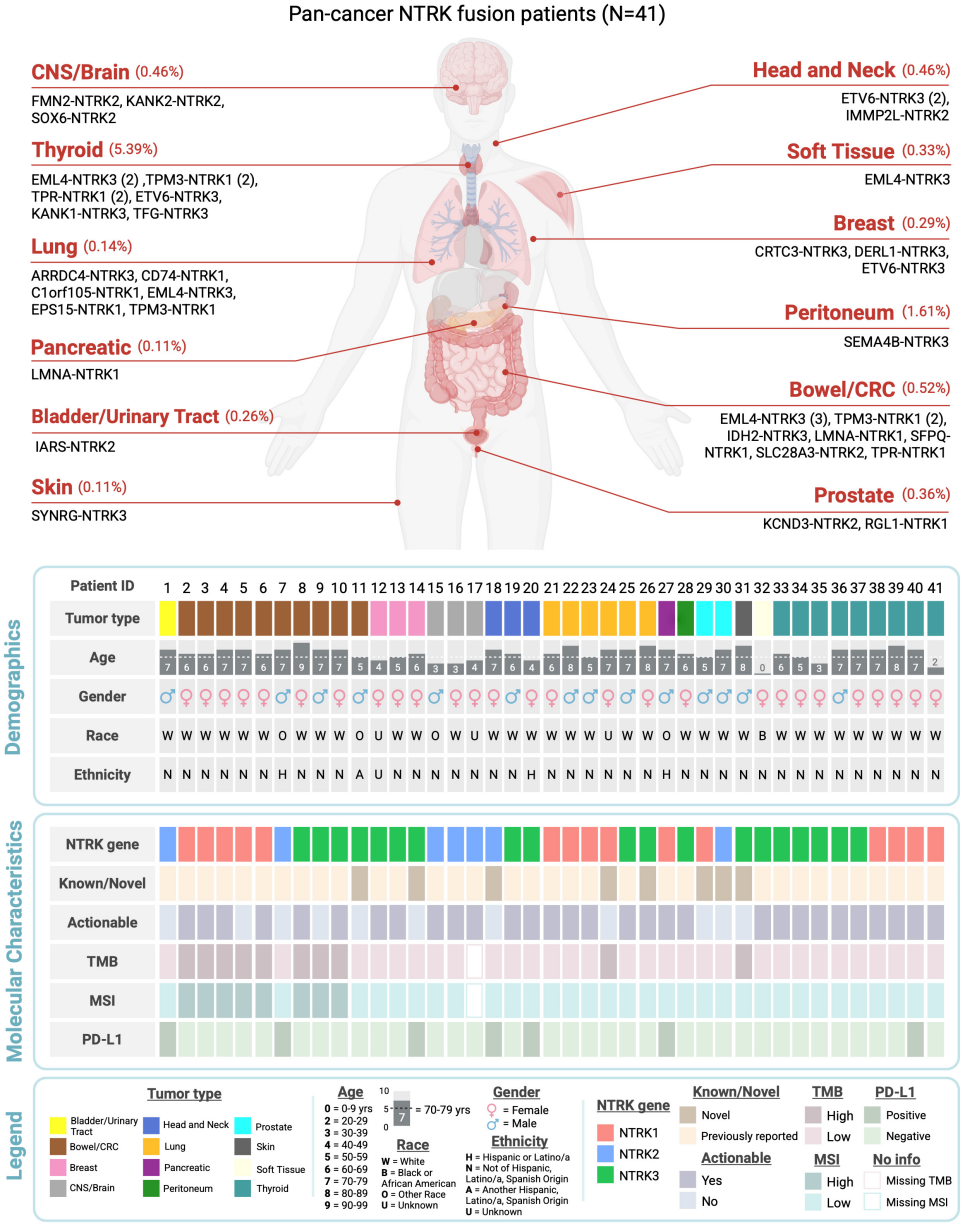
*NTRK* cohort overview (*n* = 41). Distribution of *NTRK1/2/3* gene fusions identified across individual tumor types. Demographic characteristics broken down by binned age, gender, race, and ethnicity. Molecular characteristics include *NTRK1/2/3* gene, tumor mutational burden (TMB) status, microsatellite instability (MSI) status, and PD-L1 status. Therapeutic actionability as determined by internal specifications is reported in the “Actionable” row. “Known/Novel” reports whether the fusion pair has been previous reported in the literature (known) or is novel. Missing TMB and MSI values are the results of the DNA portion of the assay failing. See legend for value specific details. Created in BioRender. Dowdell, A. (2026) https://BioRender.com/sxni8tg.

All figures were created in https://BioRender.com (Figures 1, 3A, 4) or generated in R v4.5.2 with RStudio v2025.09.2+418 using custom code snippets and utilizing *ggplot2* v4.0.1, *circlize* v0.4.17, and *ComplexHeatmap 2.26.0*.

## Results

### Prevalence of *NTRK* fusions

Tumor testing with in-house DNA and RNA hybrid capture-based CGP assays was initiated by a pathologist or oncologist immediately upon a histopathological solid tumor diagnosis for patients in this cohort (*N* = 15,128, Table 1). The median patient age was 68 years (interquartile range (IQR) 59–76), and 53% identified as female (*n* = 8,000) and 46% identified as male (*n* = 7,016). Lung cancer comprised 29% of the cohort, followed by bowel/CRC (13%), breast (7%), and skin cancer (6%) (Table 1).

**Table 1:** Overall patient cohort demographics. Patient demographics for the 15,128 patients assessed for this study. Tumor testing occurred over a period of 2018–2023.

Characteristic	Value	*N*
Total patients		15,128
Age (median [IQR])		68 [59–76]
Gender	Female	8,000
	Male	7,016
	Other/Unknown	112
Race	White	12,081
	Other/Unknown Race	1,830
	Asian	622
	Black Or African American	348
	American Indian Or Alaska Native	173
	Native Hawaiian Or Other Pacific Islander	74
Ethnicity	Not Of Hispanic, Latino/A Or Spanish Origin	13,182
	Hispanic Or Latino	856
	Unknown	1,090
Tumor type	Lung	4,376
	Bowel/CRC	1,924
	Breast	1,045
	Skin	948
	Pancreatic	907
	Esophagus/Stomach	679
	CNS/Brain	648
	Ovarian/Fallopian Tube	573
	Prostate	560
	Uterus	520
	Head and Neck	436
	Biliary Tract	387
	Bladder/Urinary Tract	384
	Soft Tissue	304
	CUP	296
	Kidney	227
	Other/Unknown	185
	Thyroid	167
	Liver	123
	Cervix	81
	Peritoneum	62
	Lymphoid	46
	Ampulla of Vater	39
	Bone	36
	Pleura	34
	Myeloid	27
	Vulva/Vagina	27
	Thymus	25
	Adrenal Gland	19
	Peripheral Nervous System	17
	Testis	15
	Eye	8
	Penis	3

*NTRK* gene fusions were detected via the RNA portion of the assay and identified 41 patients with solid tumors for an overall *NTRK* prevalence of 0.27% (0.2% pathogenic, 0.07% VUS) (Table 2, Data Sheet 1). The 41 *NTRK* fusions were detected in 12 solid tumor types, with the highest incidence in bowel/CRC (*n* = 10) and thyroid carcinoma (*n* = 9), followed by lung (*n* = 6), CNS/brain (*n* = 3), and breast cancers (*n* = 3). *NTRK1* fusions were detected in bowel/CRC (*n* = 5), thyroid (*n* = 4), lung (*n* = 4), prostate (*n* = 1), and pancreatic cancer (*n* = 1) (Figure 1). *NTRK2* fusions were detected in CNS/brain (*n* = 3), bowel/CRC (*n* = 1), head and neck (*n* = 1), bladder (*n* = 1) and prostate cancer (*n* = 1). *NTRK3* fusions were detected in thyroid (*n* = 5), bowel/CRC (*n* = 4), breast (*n* = 3), lung (*n* = 2), head and neck (*n* = 2), and peritoneum (*n* = 1), soft tissue (*n* = 1), and skin cancer (*n* = 1) (Figure 1).

**Table 2:** *NTRK* fusions detected in patient cohort. The prevalence of *NTRK* fusion cases identified in different cancer types.

Tumor type	Total cases (*N* = 15,128)	*NTRK* fusion cases (*n* = 41)	*NTRK* fusion frequency
Bladder/Urinary tract	384	1	0.26%
Bowel/CRC	1,924	10	0.52%
Breast	1,045	3	0.29%
CNS/Brain	648	3	0.46%
Head and neck	436	2	0.46%
Lung	4,376	6	0.14%
Pancreatic	907	1	0.11%
Peritoneum	62	1	1.61%
Prostate	560	2	0.36%
Skin	948	1	0.11%
Soft tissue	304	1	0.33%
Thyroid	167	9	5.39%

Total number (*N*) of cases tested, *NTRK* fusions identified, and the *NTRK* fusion frequency in the tumor type are listed.

### Known and novel *NTRK* gene fusion partners

**Figure 2:**
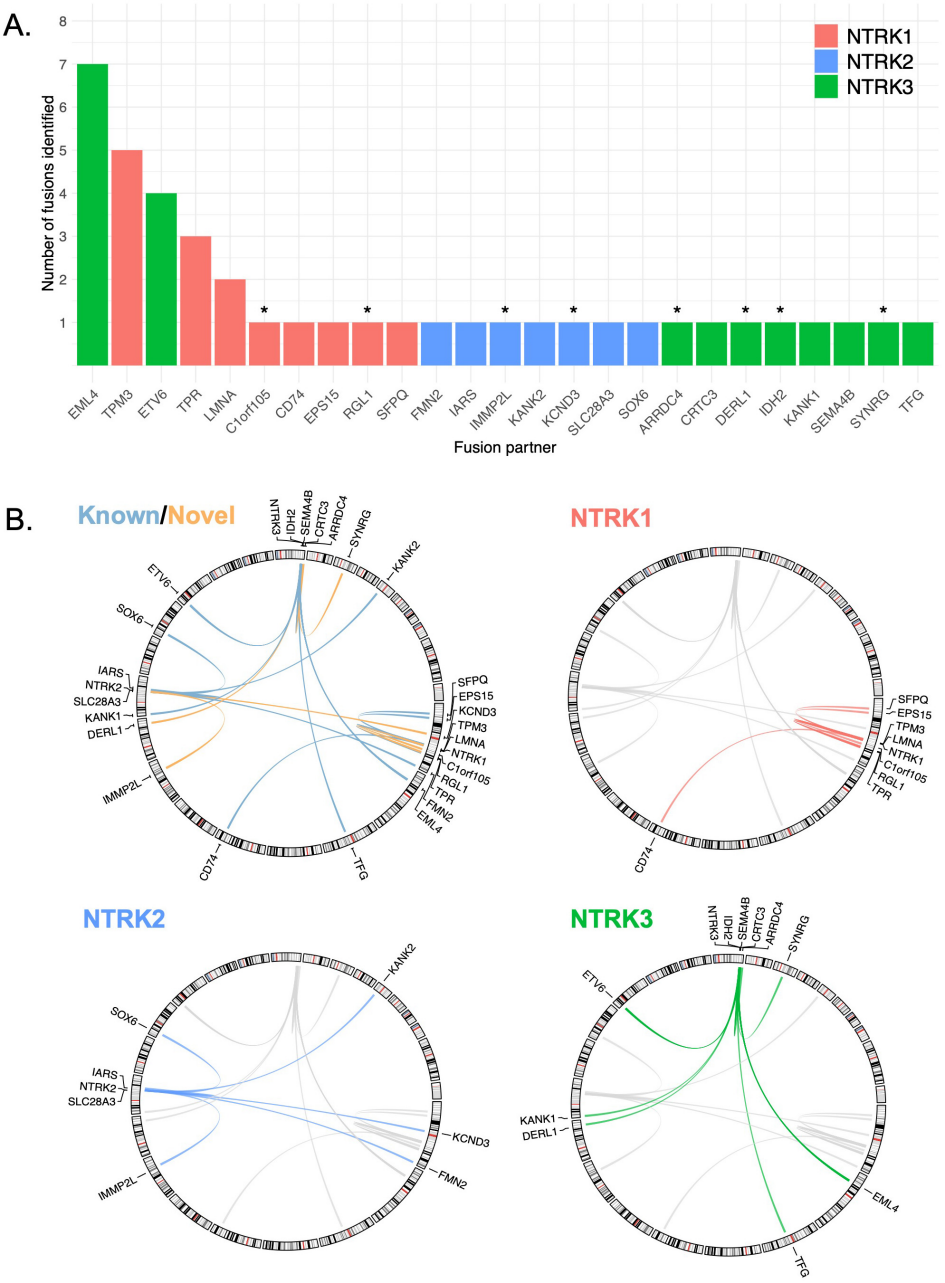
Diversity of *NTRK* fusion gene partners identified in cohort. **(A)** Frequency of unique fusion partners identified across *NTRK* genes. * Denotes novel fusions previously unreported in public databases. **(B)** Circos plots depicting known (blue) vs. novel (orange) fusion pairs as well as colored to highlight *NTRK1/2/3* respectively. Gray lines are links representing *NTRK* fusion pairs from our cohort with an *NTRK* driver gene (*NTRK1/2/3*) not currently being highlighted (i.e., in *NTRK1* circos plot, gray lines represent *NTRK2/3* fusion pairs).

We identified 25 unique *NTRK* fusion partner pairs among the 41 patients harboring *NTRK* fusions (Data Sheet 1). *EML4* (*n* = 7), *TPM3* (*n* = 5), and *ETV6* (*n* = 4) were the most frequently identified *NTRK* fusion partners (Figure 2A). 32% (*n* = 8/25) of fusion partners were not previously reported in other large public databases/studies and were identified with *NTRK1* (*n* = 2), *NTRK2* (*n* = 2), and *NTRK3* (*n* = 4). Novel partners included *IDH2, DERL1, IMMP2L, C1orf105, RGL1, KCND3, SYNRG*, and *ARRDC4* (Figure 2). Novel fusions were identified across tumor types, including prostate and lung cancer (*n* = 2), in addition to head and neck, breast, bowel/CRC, and skin cancer (*n* = 1) (Figure 1).

**Figure 3:**
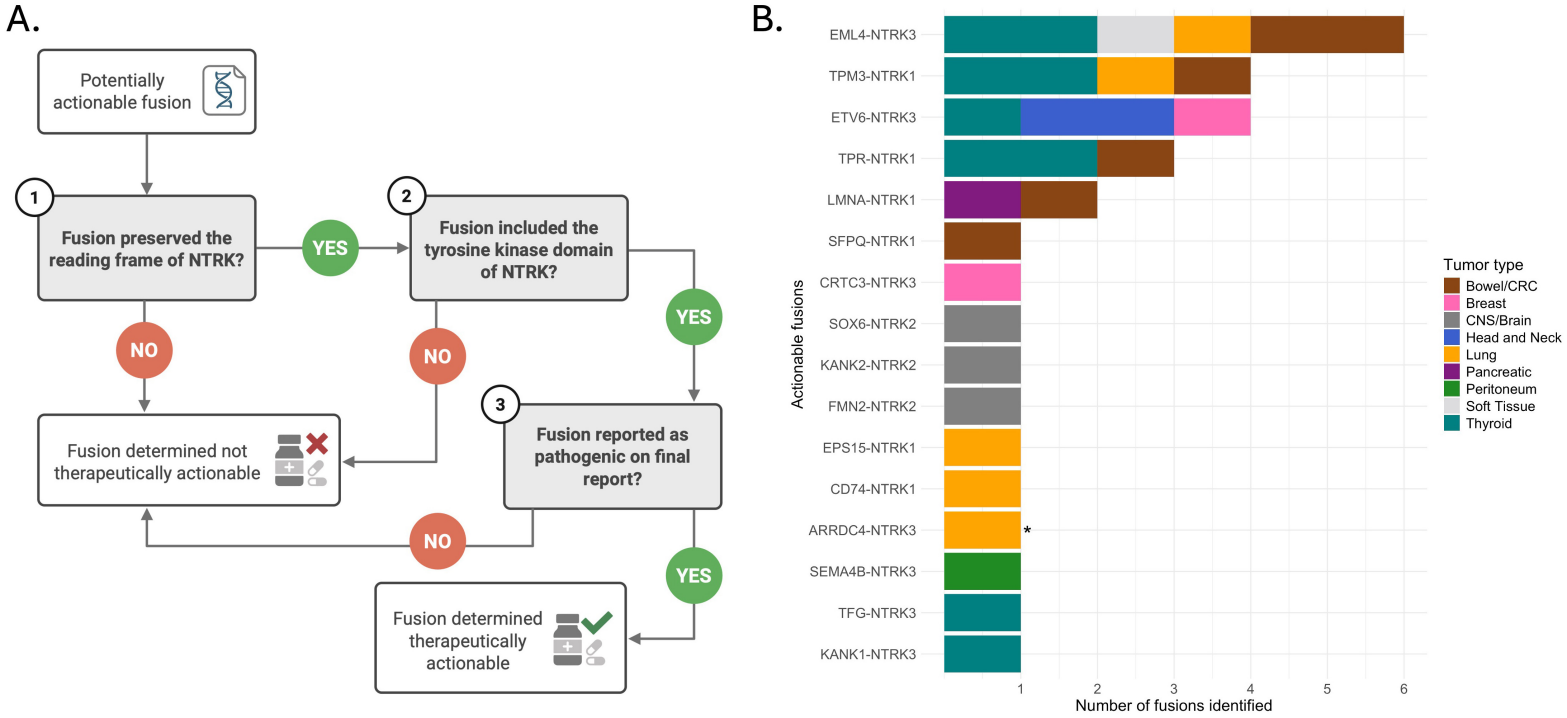
Therapeutically actionable *NTRK* fusions and criteria used to determine actionability. **(A)** Flowchart depicting therapeutic actionability assessment for *NTRK* fusions. The three decision points shown correspond directly to the criteria described in the Methods section: (1) whether the fusion preserves the *NTRK* reading frame, (2) whether the fusion includes the *NTRK* kinase domain; and (3) whether the fusion is reported on the final CGP report as pathogenic. Fusions failing any criterion are categorized as variants of unknown significance (VUS) and considered not therapeutically actionable. Discretionary supplemental testing (e.g., pan-TRK IHC) was occasionally used to adjudicate novel or borderline cases; fusions not confirmed by such testing remained VUS. Created in BioRender. Dowdell, A. (2026) https://BioRender.com/srjsmt7. **(B)** The number of therapeutically actionable *NTRK* fusions identified in each tumor type. *Denotes novel fusion previously unreported in public databases.

In our cohort, 73% (*n* = 30/41) of the detected *NTRK* fusions were determined to be likely therapeutically actionable and reported as pathogenic (Figure 3). *NTRK* fusions were deemed therapeutically actionable if the fusion preserved the reading frame of *NTRK*, included the tyrosine kinase domain of *NTRK*, and were included on the Providence CGP testing report as pathogenic (Figure 3A). The 11 *NTRK* gene fusions that did not have these features were reported in the variants of unknown significance (VUS) section of the Providence CGP testing report. Actionable fusions were identified across *NTRK* gene and tumor type, including thyroid (*n* = 9), bowel/CRC (*n* = 6), and lung cancer (*n* = 5) (Figure 3B). The most common *NTRK* fusions reported as actionable included *EML4-NTRK3* (*n* = 6), *ETV6-NTRK3* (*n* = 4), and *TPM3-NTRK1* (*n* = 4). One novel fusion (*ARRDC4-NTRK3*) was determined to be therapeutically actionable and reported in lung cancer (Figure 3B).

### Co-occurrence of *NTRK* gene fusions with other actionable alterations

**Figure 4:**
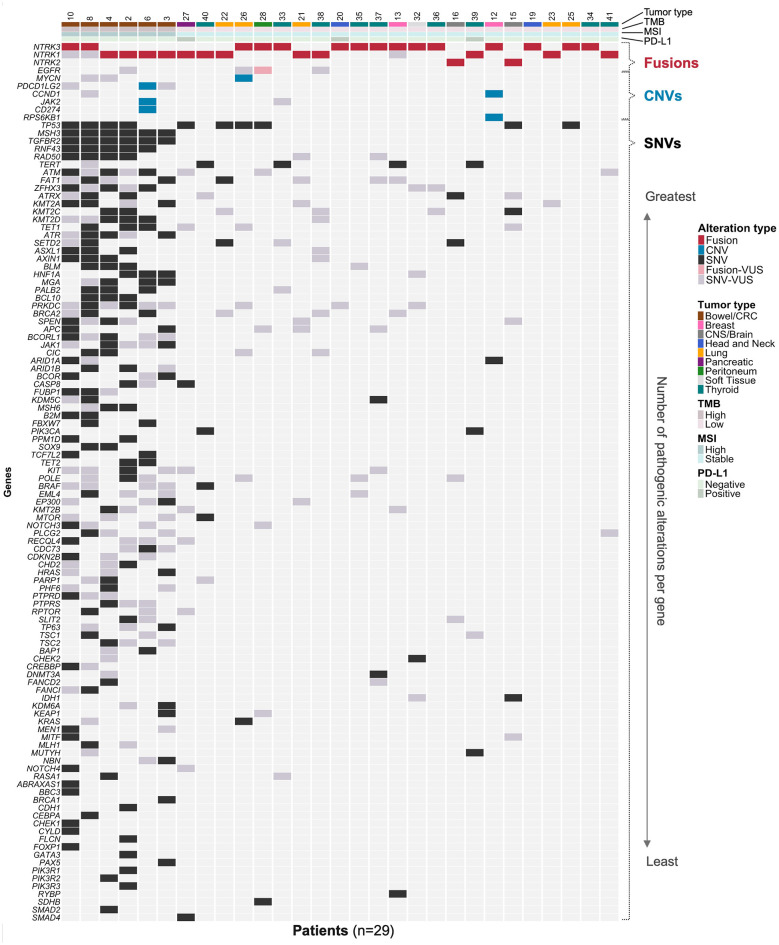
Co-occurring alterations plot of therapeutically actionable *NTRK* fusions. For each patient with an actionable *NTRK* fusion (*n* = 29), the all co-occurring pathogenic alterations detected by the assay are shown. Patient columns are annotated with tumor type, tumor mutational burden (TMB), microsatellite instability (MSI) status, programmed death-ligand 1 (PD-L1) status, and the *NTRK* gene involved. Rows display fusions, copy-number variants (CNVs), small variants (SNPs and indels), and variants of unknown significance (VUS). Genes included in the heatmap were limited to those with at least one pathogenic variant reported for the subset of patients (122 of 396 genes). Rows are ordered by biomarker class and then by decreasing frequency of pathogenic variants within each class; genes with equal pathogenic variant frequency are secondarily ordered by VUS frequency. Created in BioRender. Dowdell, A. (2026) https://BioRender.com/xaxn6cy.

Co-occurrence of *NTRK* gene fusions with other actionable alterations was rare with the exception in bowel/CRC patients (Figure 4, Data Sheet 1). Among the 30 cases harboring a potentially actionable *NTRK* fusion, 29 patients had successful DNA sequencing. Among the 29 patients we could evaluate for co-occurring alterations, 79% (*n* = 23/29) did not have any co-occurring OncoKB Levels of Evidence 1 or 2 therapeutic alteration, which would correspond to AMP/ASCO/CAP Level A evidence for FDA approved therapies (24). Additionally, 31% (*n* = 9/29) of cases had no other reported pathogenic alterations (Figure 4, Data Sheets 1, 2). We observed all bowel/CRC patients with an actionable *NTRK* fusion (*n* = 6) were also MSI-H and TMB-H. Co-occurrence between *NTRK* fusion positivity and MSI-H and TMB-H was statistically significant in bowel/CRC (*p* < 0.001). In contrast, no statistically significant association between *NTRK* fusion positivity and MSI-H or TMB-H was observed across tumor histologies. PD-L1 expression was positive in 3 different tumor types, including thyroid (*n* = 1), head and neck (*n* = 1), and pancreatic cancer (*n* = 1). *BRAF* p.V600E substitution in thyroid cancer and *IDH1* mutation in glioma were the only co-alterations with therapeutic implications in the cohort (Figure 4, Data Sheet 1).

## Discussion

Testing for *NTRK* fusions has become pivotal in clinical practice due to the availability of highly selective TRK inhibitors that have demonstrated improved response rates and quality of life of patients (15, 17, 25). This study, involving 15,128 patients from the Providence community health system, identified an overall 0.27% prevalence of *NTRK* fusions (0.2% pathogenic, 0.07% VUS) across 12 different tumor types, underscoring the broad applicability and necessity of comprehensive genomic profiling (CGP). This study corroborates other research analyzing *NTRK* prevalence in large real-world cohorts from academic centers and commercial labs (11, 12, 23, 26). Notably, our *NTRK* prevalence aligns closely with the pan-cancer observations at Memorial Sloan Kettering (MSK) reporting a 0.28% *NTRK* fusion prevalence across ~26,000 prospectively sequenced patients (using DNA-based MSK-IMPACT with reflex RNA testing via MSK-Fusion), and falls within a similar range of the 0.20% reported in a nationwide Japanese CGP registry (C-CAT) which observed higher prevalence in pediatric than adult cases (*n* = 46,421) (12, 27). Moreover, a large U.S. reference-laboratory series of send-out CGP tests (*n* = 19,591) utilizing concurrent DNA+RNA hybrid-capture reported a pan-cancer *NTRK* fusion prevalence of 0.35% (28). A recent systematic review similarly places pan-cancer prevalence generally below 0.5% and highlights that RNA-inclusive methods (RNA or combined DNA/RNA assays) detect more fusions than DNA-only approaches, consistent with our use of a DNA/RNA hybrid-capture panel (29). Despite differences in ancestry, pediatric representation, and tumor type mix, these concordant estimates across academic (MSK), national registry (Japan), and our community health setting support the generalizability of a ~0.2–0.35% pan-cancer prevalence for *NTRK* fusions. Contextually, the tumor-agnostic activity of larotrectinib reported across adult and pediatric populations reinforces the clinical rationale for broad *NTRK* fusion testing (14).

*NTRK* fusions were identified across multiple tumor types, including bowel/CRC, thyroid, and lung cancer, which were the tumor types most frequently tested for *NTRK* fusions in a recent survey of community-based medical oncologists (30). This highlights the clinical importance of identifying *NTRK* fusions across diverse solid tumor types through DNA and RNA hybrid capture-based next-generation sequencing (NGS) assays. We instituted a pathologist-directed or reflex testing model where CGP testing is initiated by a pathologist at the diagnosis of solid tumors. Reflex testing decreases test failures and reduces non-biomarker informed care enabling all patients with cancer to get tested with CGP (31). This approach likely contributed to the identification of *NTRK* fusions across the broad spectrum of tumor types observed in this study. Methodologically, our combined DNA/RNA panel, ProvSeq 523 falls in line with strategies used in large academic centers, where DNA-suggestive events are reflexed to RNA for confirmation to minimize inclusion of fusions of unknown significance while maintaining what is feasible within our large community health system (12, 20).

Additional studies are warranted to assess the value of reflex testing for identifying patients that may benefit from a precision medicine approach. Notably, 73% of the detected fusions were deemed therapeutically actionable, emphasizing the critical role of *NTRK* fusion testing in guiding treatment decisions, especially with the availability of effective TRK inhibitors like larotrectinib and entrectinib. *NTRK* fusions were the only OncoKB Therapeutic Level 1 or Level 2 alteration reported in 79% of *NTRK* fusion-positive cases, highlighting the importance of broad biomarker testing. These findings are consistent with large real-world and institutional datasets in which confirmed *NTRK* fusions are uncommon but broadly distributed across histologies and often occur in tumors with few alternative oncogenic drivers (12).

Recommendations for *NTRK* testing have been provided, many of which recommend increased *NTRK* testing and discuss the different types of testing methodologies (18–20, 32). Different testing algorithms have been proposed, mostly driven by the frequency of *NTRK* fusions identified in the tumor type. For example, the ESMO Translational Research and Precision Medicine Working Group recommends using FISH, RT-PCR, or targeted RNA NGS in tumor types known to harbor *NTRK* fusions, and IHC or NGS in tumor types without recurrent *NTRK* fusions (19). These consensus statements emphasize that in unselected populations where *NTRK* fusions are rare either front line CGP with an RNA component or an IHC screening with sequencing follow up approach is appropriate to ensure sensitivity and specificity. *NTRK* genes are highly promiscuous with fusion partners, with over 60 reported 5′ binding partners (33). This study yielded similar results to prior studies showing that *EML4, TPM3*, and *ETV6* were the most common partner genes in *NTRK* fusion-positive cancers (1). Additionally, this study identified 8 novel *NTRK* fusion partners (*IDH2, DERL1, ARRDC4, SYNRG, C1orf105, IMMP2L, KCND3, RGL1*), underscoring the advantages of RNA-based NGS in identifying both known and novel *NTRK* fusions, as one study observed RNA NGS identifying *NTRK* fusions in 26% of cases missed by DNA-only approaches (12).

Moreover, in the bowel/CRC cohort in this study, all actionable *NTRK* fusions were identified in MSI-H tumors. *NTRK* fusions are rare in CRC, with rates of 0.2% to 1%, but increases to around 5% in MSI-H tumors (11, 34). The enrichment of *NTRK* fusions in MSI-H bowel/CRC is concordant with large academic centers and supports routine MSI and fusion assessment in bowel/CRC to guide therapy (12, 28). In this unique scenario, understanding the full genomic picture is important for genetic testing and therapeutic selection, as both immunotherapy and targeted therapy are options for MSI-H and *NTRK* fusion-positive CRC. Thus, this study supports the routine implementation of CGP in clinical practice to capture novel fusions and co-occurring alterations in *NTRK* fusion-positive tumors.

TRK inhibitors have demonstrated activity in tumors harboring *NTRK* fusions regardless of *NTRK* gene or fusion partner (14–17). In this study, 30 out of the 41 identified *NTRK* fusions were identified as actionable. The 11 *NTRK* fusions were considered not actionable due to a frameshift of *NTRK* (or the reading frame being undeterminable due to an intronic breakpoint of *NTRK*), exclusion of the kinase domain, or in one unique instance the *NTRK* fusion for patient #5 was sent for IHC confirmatory testing at the discretion of the reviewing pathologist due to borderline sequencing QC metrics, but was not confirmed and remained a VUS on the Providence CGP test report. Accurate variant interpretation is becoming increasingly complex, and resources are emerging to support curation and informed decisions for *NTRK* fusion-positive tumors (35). Recently, an evidence-based scoring framework was developed for assessing the oncogenicity of *NTRK* fusions using three primary specifications: fusion structure and reading frame, cancer association, and functional evidence (35).

Taken together, these data contribute to the growing body of evidence highlighting that testing *NTRK* fusions across tumor types is critical, as these patients often lack other actionable biomarkers in their tumors. DNA and RNA sequencing reveals the complete genomic landscape of *NTRK* positive fusions and can help guide appropriate therapy selection and sequencing. Furthermore, DNA/RNA CGP studies across diverse health systems consistently converging on pan-cancer *NTRK* fusion prevalence of ~0.2%−0.35% with higher rates being found in rare histologies and MSI-H bowel/CRC reinforce the value in broad reflex-enabled testing strategies in routine clinical care.

## Data Availability

The original contributions presented in the study are included in the article/Supplementary material, further inquiries can be directed to the corresponding author.
